# CITF1 Functions Downstream of SPL7 to Specifically Regulate Cu Uptake in *Arabidopsis*

**DOI:** 10.3390/ijms23137239

**Published:** 2022-06-29

**Authors:** Yuerong Cai, Gang Liang

**Affiliations:** 1 CAS Key Laboratory of Tropical Plant Resources and Sustainable Use, Xishuangbanna Tropical Botanical Garden, The Innovative Academy of Seed Design, Chinese Academy of Sciences, Kunming 650223, China; caiyuerong@xtbg.ac.cn; 2 The College of Life Sciences, University of Chinese Academy of Sciences, Beijing 100049, China

**Keywords:** CITF1, SPL7, Copper, FRO4, FRO5, COPT2, *Arabidopsis*

## Abstract

Copper (Cu) is one of the most indispensable micronutrients, and proper Cu homeostasis is required for plants to maintain essential cellular functions. Plants activate the Cu uptake system during Cu limitation. Although SPL7 (SQUAMOSA PROMOTER BINDING PROTEIN-LIKE 7) and CITF1 (Cu-DEFICIENCY INDUCED TRANSCRIPTION FACTOR 1) are two transcription factors in Cu homeostasis, it remains unclear how SPL7 and CITF1 control the Cu uptake system. Here, we reveal that overexpression of *CITF1* causes the enhanced tolerance to Cu deficiency and the elevated expression of Cu uptake genes *COPT2*, *FRO4* and *FRO5*. Electrophoretic mobility shift assays (EMSA) and transient expression assays indicate that SPL7 directly binds to and activates the promoter of *CITF1*. The overexpression of *CITF1* partially rescues the sensitivity of *spl7-1* to Cu deficiency. Transcriptome data suggest that SPL7 and CITF1 coregulate the Cu-homeostasis-signaling network, and CITF1 has its own independent functions. Moreover, both SPL7 and CITF1 can directly bind to and activate the promoters of three Cu uptake genes *COPT2*, *FRO4* and *FRO5*. This work shows the functions of CITF1 in the Cu-homeostasis-signaling network, providing insights into the complicated molecular mechanism underlying Cu homeostasis.

## 1. Introduction

Copper (Cu) is one of the mineral nutrients in plants and acts as a redox active cofactor in a wide variety of plant proteins such as plastocyanin, cytochrome c oxidase, Cu/Zn superoxide dismutase (CSD), ethylene receptors, laccases, ascorbate and amine oxidases, plantacyanin, polyphenol oxidases, etc. These proteins are essential for fundamental biological processes in plants, including photosynthetic and respiratory electron transport, oxidative stress protection, cell wall metabolism, ethylene perception, pathogen defense, and molybdenum cofactor biosynthesis [1,2,3]. Therefore, Cu is a vital trace element for the growth and development of plants.

In soil, Cu ions are prone to binding soil components and can be absorbed onto the surfaces of clays and Fe or Mn oxides, precipitated with carbonates and phosphates, or sequestered in silicate minerals [4]. Thus, plants often face Cu deficiency. When suffering from Cu deficiency, plants develop Cu-deficiency symptoms such as curled leaf margins, reduced fertility, abnormal fruit or seed formation, and yield damage [1,5,6,7,8]. Although Cu is indispensable for plants, excessive Cu reacts in cells with oxygen and generates noxious reactive oxygen species that are deleterious for plant growth and development [9]. Consequently, plants have to maintain Cu levels in a proper range, namely Cu homeostasis.

Certain plants have evolved sophisticated mechanisms to efficiently acquire and utilize Cu. In response to Cu limitation, these plants economize Cu by modifying Cu allocation for the efficient utilization of that limited resource. For example, *Arabidopsis* plants switch their types of superoxide dismutase (SOD) to adapt to Cu deficiency; the Cu-containing CSD1 and CSD2 are downregulated, and their function is replaced by Fe SOD under Cu deficiency conditions [5]. Another type of Cu-containing proteins are laccases, some of which are downregulated in order to reduce Cu consumption in response to Cu limitation. Additionally, these plants mobilize the limited Cu between tissues. Under Cu deficiency conditions, a Cu chaperone, CCH (Copper Chaperone), is induced and may be involved in Cu translocation during senescence [10]. In addition to economically utilizing Cu, these plants can activate a high-affinity Cu uptake system to elevate Cu uptake under Cu deficiency conditions. The Cu uptake system includes Cu^2+^-reductases FERRIC REDUCTION OXIDASE 4 (FRO4) and FRO5 and high-affinity Cu transporters (COPT1, COPT2, and COPT6) at the plasma membrane that mediate Cu transport to the cytoplasm [11,12,13,14].

In *Arabidopsis*, SPL7 (SQUAMOSA PROMOTER BINDING PROTEIN-LIKE 7) plays a key role in reprogramming gene expression in order to promote Cu economization and uptake under Cu-deficiency conditions [12,15]. In Cu-deficient plants, SPL7 upregulates the abundance of Cu-miRNAs, *miR398*, *miR397*, *miR408*, and *miR857*, which in turn cause the reduction of their target transcripts, such as *CSD1*, *CSD2*, and several *LACs* (LACCASE genes encoding laccases) [12,15,16,17]. To compensate for the decline of CSDs, SPL7 activates the expression of *Fe SOD 1* (*FSD1*). SPL7 is also in charge of the upregulation of Cu uptake genes during Cu limitation [12,15]. KIN17 was reported to cooperate with SPL7 to maintain Cu homeostasis [18].

Cu-DEFICIENCY INDUCED TRANSCRIPTION FACTOR1 (CITF1, also known as bHLH160) is a Cu-deficiency-induced transcription factor which regulates Cu uptake into roots and its delivery to flowers, affects jasmonic acid synthesis, and is required for normal plant growth under Cu deficiency [19]. In the present study, we further characterized the functions of CITF1 in regulating Cu uptake. We found that CITF1 is a direct target of SPL7 under Cu-deficiency conditions. Our data indicated that SPL7 and CITF1 coregulate the Cu-signaling network and are required for the full activation of Cu-deficiency responses. We also suggested that CITF1 and SPL7 activate the Cu uptake system by directly regulating the expression of *COPT2*, *FRO4,* and *FRO5*.

## 2. Results

### 2.1. CITF1 Specifically Affects the Expression of Cu Uptake Genes COPT2, FRO4 and FRO5

To further investigate how CITF1 regulates Cu uptake, the homozygous *citf1-1* mutant [19] was isolated and the complementation lines (COM1 and COM2) of *citf1-1* (Figure S1) were constructed. Although no visible phenotypic difference was observed between *citf1-1* and the wild type under +Cu (1µM CuSO4) conditions, *citf1-1* developed shorter roots than the wild type under −Cu (Cu-free) conditions (Figure S1A,B). Meanwhile, the complementation lines grew as well as those of the wild type. Next, the rosettes from plants grown in soil were harvested and used for Cu-concentration analysis. Significantly, the Cu concentration of *citf1-1* was lower than that of the wild type and the complementation lines (Figure S1C), consistent with the previous report [19].

**Figure 1:**
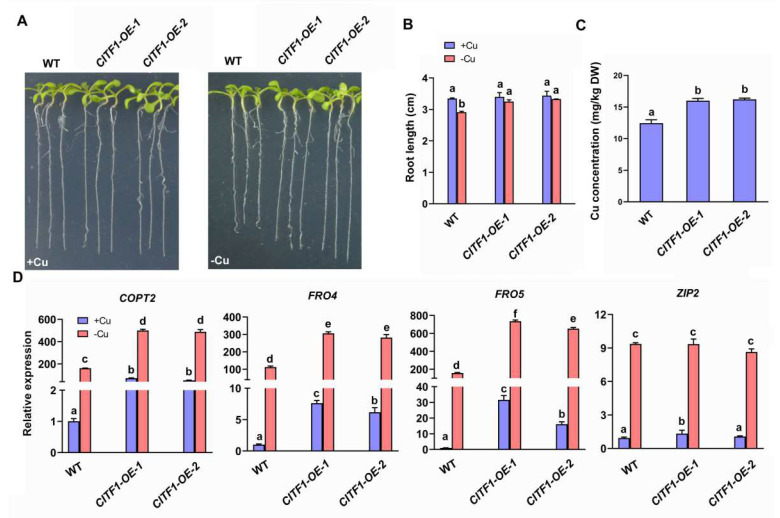
Overexpression of *CITF1* enhances Cu uptake. (**A**) Seedlings were grown on +Cu or −Cu medium for 7 days. (**B**) Root length. Seven-day-old seedlings were analyzed. Three biological replicates were conducted. Data represent arithmetic means ± SD (*n* = 3 biological replicates, each of which contained at least 15 plants). Different letters above each bar indicate statistically significant differences (ANOVA, *p* < 0.01). (**C**) Cu concentration. Leaves from plants grown in soil for 3 weeks were used for Cu measurement. Data represent arithmetic means ± SD (*n* = 3 biological replicates, each of which contained 100 mg pooled dry leaves). Different letters above each bar indicate statistically significant differences (ANOVA, *p* < 0.01). (**D**) Expression of Cu-deficiency responsive genes. Seedlings were grown on +Cu or −Cu medium for 7 days. Roots were used for RNA extraction. Data represent arithmetic means ± SD (*n* = 3). Different letters above each bar indicate statistically significant differences (ANOVA, *p* < 0.01).

Next, we asked whether the elevated expression of *CITF1* would enhance the Cu-deficiency response. Thus, we constructed transgenic plants (*CITF1-OE*) constitutively expressing HA-tagged CITF1. Subsequently, we tested their growth on +Cu and −Cu media, respectively. The growth of *CITF1-OE* plants was similar to the wild type when grown on +Cu medium. In contrast, compared with the wild type, the *CITF1-OE* plants displayed an enhanced tolerance to Cu deficiency, as shown by the longer roots (Figure 1A,B). Correspondingly, we also measured Cu concentration, finding that the *CITF1-OE* plants had higher Cu concentration than the wild type (Figure 1C). These results suggest that the overexpression of *CITF1* promotes the Cu accumulation.

We determined that the expression of Cu-deficiency-responsive genes, such as *COPT2*, *FRO4*, *FRO5*, *ZIP2*, *YSL2,* and *FSD1*. *COPT2*, *FRO4,* and *FRO5* dramatically decreased in the *citf1-1* under Cu-deficiency conditions (Figure S2), supporting the previous report [19]. In contrast, *ZIP2*, *YSL2,* and *FSD1* did not change significantly (Figure S2). Correspondingly, the expression of *COPT2*, *FRO4,* and *FRO5* was considerably upregulated compared with the wild type, regardless of Cu status (Figure 1D). These data suggest that CITF1 specifically affects the expression of Cu uptake genes *COPT2*, *FRO4,* and *FRO5*.

### 2.2. CITF1 Is a Direct Target of SPL7

**Figure 2:**
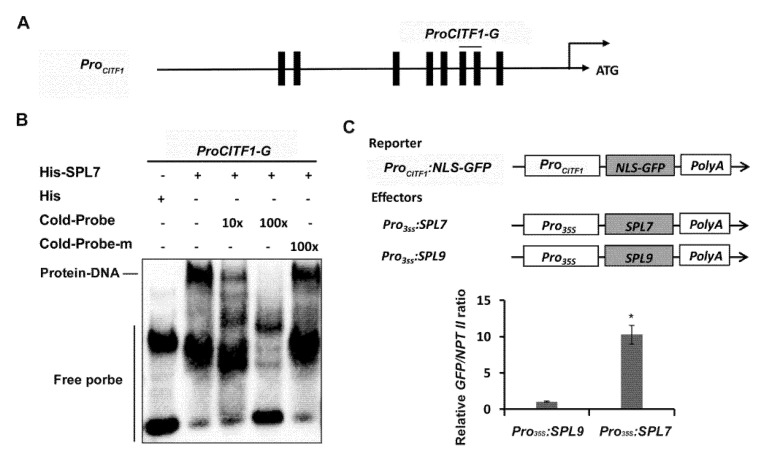
SPL7 directly activates *CITF1.* (**A**) GTAC-boxes are indicated in the *CITF1* promoter. (**B**) EMSA showing that SPL7 directly binds to the promoter of *CITF1*. Either His-SPL7 or 6×His was incubated with the biotin-labeled probes. Biotin-probe, biotin-labeled probe; cold-probe, unlabeled probe; cold-probe-m, unlabeled mutated probe with mutated GTAC-boxes. (**C**) Activation of *CITF1* promoter by SPL7. The *CITF1* promoter was used to drive the NLS-GFP as the reporter. *SPL7* and *SPL9* driven respectively by the CaMV 35S promoter were used as the effectors. The SPL9 was used as a negative control effector. The *GFP/NPT II* ratio represents the *GFP* transcript abundance relative to the internal control *NPT II* transcript. Data represent arithmetic means ± SD (*n* = 3). * *p* < 0.01.

It was reported that *CITF1* is induced by Cu deficiency [12,19]; however, it is unclear if SPL7 directly regulates the expression of *CITF1*. As an SBP-domain-containing protein, SPL7 recognizes GTAC cis elements in its target promoters [15,20]. Sequence analysis indicated that there are eight GTAC boxes in the *CITF1* promoter (Figure 2A). We proposed that SPL7 directly binds to the *CITF1* promoter by recognizing the GTAC boxes. To confirm this hypothesis, we conducted electrophoretic mobility shift assays (EMSAs). Since the SBP domain of SPL7 is responsible for DNA binding, the SPL7 N-terminal containing the SBP domain was fused with the 6×His tag and expressed in and purified from Escherichia coli. A DNA fragment with two GTAC boxes was used as the probe in EMSAs. The recombinant His-SPL7 protein dramatically reduced the electrophoretic mobility of biotin-labeled probes, resulting in a super-shift band. The unlabeled wild-type probes (Cold-Probe) reduced the binding of biotin-labeled probes with SPL7, whereas the cold mutated probes (Cold-Probe-m) with the mutated GTAC boxes did not (Figure 2B).

To further determine whether SPL7 activates the expression of *CITF1*, we performed transient expression assays. The promoter region of *CITF1* was used to drive a nuclear localization signal (NLS)-fused *GFP* gene as the reporter. The full-length coding sequences of SPL7 and SPL9 were respectively driven by the 35S promoter as the effectors, and SPL9 was used as the control effector (Figure 2C). The reporter was co-expressed with the effectors *Pro_35S_:SPL9* and *Pro_35S_:SPL7*, respectively, in tobacco leaves, and then the expression of GFP was examined. Compared with the control effector *Pro_35S_:SPL9*, *Pro_35S_:SPL7* significantly activated the promoter of *CITF1* (Figure 2C). Taken together, our data suggest that SPL7 directly activates the expression of *CITF1*.

### 2.3. Genetic Interaction of CITF1 and SPL7

**Figure 3:**
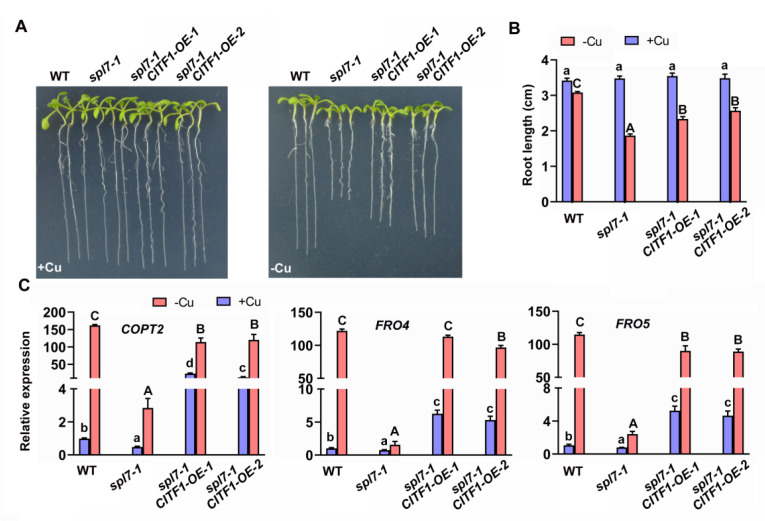
Overexpression of *CITF1* partially rescues *spl7-1*. (**A**) Phenotypes of seedlings. Seedlings grown on +Cu or −Cu medium for 7 days are shown. (**B**) Root length. Seven-day-old seedlings were analyzed. Three biological replicates were conducted. Data represent arithmetic means ± SD (*n* = 3 biological replicates, each of which contained at least 15 plants). Different letters above each bar indicate statistically significant differences (ANOVA, *p* < 0.01). (**C**) Expression of Cu-deficiency responsive genes. Seedlings were grown on +Cu or −Cu medium for 7 days. Roots were used for RNA extraction. Data represent arithmetic means ± SD (*n* = 3). Different letters above each bar indicate statistically significant differences (ANOVA, *p* < 0.01).

Given that *CITF1* is involved in the regulation of Cu homeostasis and is a target of SPL7, we sought to test whether the overexpression of *CITF1* could rescue the defective response of *spl7-1* to Cu-deficiency. The *CITF1* overexpression construct was introduced into the *spl7-1* mutant, and the transcript abundance of *CITF1* was examined (Figure S3). Subsequently, plants were grown on −Cu medium for phenotype analysis. As expected, the overexpression of *CITF1* partially rescued the sensitivity of *spl7-1* to Cu deficiency (Figure 3A,B). Meanwhile, the expression of *COPT2*, *FRO4,* and *FRO5* was dramatically increased in the *spl7-1/CITF1-OE* plants compared with the *spl7-1* mutant (Figure 3C). These results suggest the partial function complementation of *spl7-1* by the overexpression of *CITF1*.

**Figure 4:**
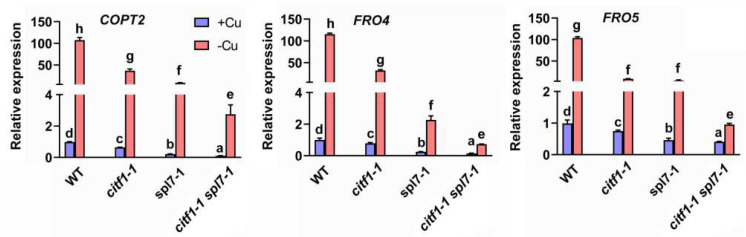
Expression of Cu uptake genes in *citf1-1 spl7-1*. Seedlings were grown on +Cu or −Cu medium for 7 days. Roots were used for RNA extraction. Data represent arithmetic means ± SD (*n* = 3). Different letters above each bar indicate statistically significant differences (ANOVA, *p* < 0.01).

To further uncover the genetic relationship of SPL7 and CITF1 in Cu-deficiency response, we constructed the *citf1-1 spl7-1* double mutants by crossing the corresponding *citf1-1* and *spl7-1* mutants (Figure S4A–C). When grown on +Cu medium, neither the single mutants nor the double mutant showed any obvious phenotypic differences compared with wild type plants. In contrast, when exposed to −Cu medium, all mutants displayed Cu-deficient symptoms, and the double mutant had the severest phenotypes (Figure S4D). Similarly, when grown in hydroponic solution, the growth of *citf1-1 spl7-1* was severely retarded (Figure S4E). We then determined the expression of their common target genes, *COPT2*, *FRO4,* and *FRO5*. In consistence with the phenotypes, the *citf1-1 spl7-1* mutant had the lowest levels of *COPT2*, *FRO4,* and *FRO5* compared with *citf1-1* and *spl7-1* (Figure 4). Taken together, our data suggest that CITF1 functions downstream of SPL7, and it plays an additive role in the regulation of Cu uptake genes.

### 2.4. CITF1 and SPL7 Coregulate Cu-Deficiency Response

**Figure 5:**
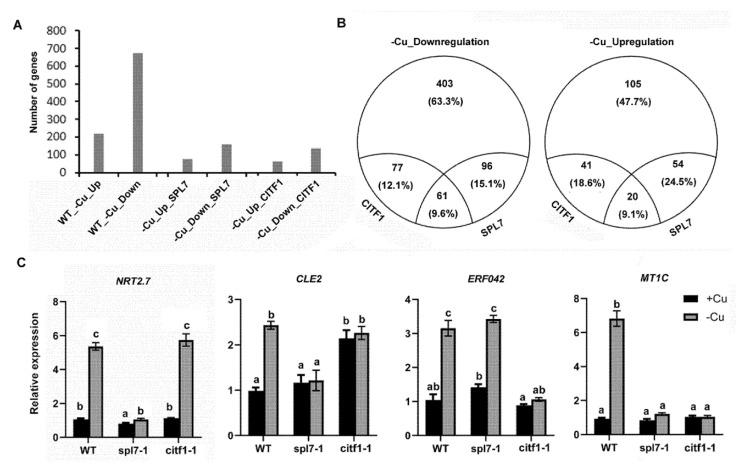
Effects of CITF1 and SPL7 on the expression of Cu deficiency responsive genes. (**A**) Genes responding transcriptionally to Cu deficiency. WT_−Cu_Up, genes upregulated by −Cu in wild type; WT_−Cu_Down, genes genes downregulated by −Cu in wild type; −Cu_Up_SPL7, genes upregulated dependently of SPL7; −Cu_Down_SPL7, genes downregulated dependently of SPL7; −Cu_Up_CITF1, genes upregulated dependently of CITF1; −Cu_Down_CITF1, genes downregulated dependently of CITF1. (**B**) Pie chart indicating percentages of coregulation by CITF1 and SPL7. (**C**) Validation of some differentially expressed genes. Seedlings were grown on +Cu or −Cu medium for 7 days. Roots were used for RNA extraction. Data represent means ± SD (*n* = 3). Different letters above each bar indicate statistically significant differences (ANOVA, *p* < 0.01).

We have confirmed that CITF1 plays a key role in the maintenance of Cu homeostasis. Given that SPL7 and CITF1 are two important transcription factors, we wondered how they control the Cu-deficiency response network. To dissect the roles of CITF1 and SPL7 in controlling transcription during Cu depletion, we performed transcriptional profiling of the wild type, *citf1-1*, and *spl7-1* in response to Cu-deficiency. We identified 220 genes responding to Cu deficiency with an at least twofold upward change in transcript levels, and 637 genes with an at least twofold downward change, and we designated them Cu-deficiency upregulated (−Cu_Up) and downregulated (−Cu_Down) genes, respectively (Figure 5A; Table S1). Among those −Cu_Up genes, the transcript abundance of 74 genes is at least twofold lower in the *spl7-1* than in the wild type under −Cu conditions, and they are named SPL7 dependent −Cu_Up (−Cu_Up_SPL7) genes. Of the −Cu_Down genes, 157 genes, whose transcript abundance is at least two-fold higher in the spl7-1 than in the wild type under −Cu conditions, are named SPL7 dependent −Cu_Down (−Cu_Down_SPL7) genes. By a similar filtration, we identified 61 CITF1 dependent −Cu_Up (−Cu_Up_CITF1) genes and 138 CITF1 dependent −Cu_Down (−Cu_Down_CITF1) genes. We further analyzed and compared the SPL7 and CITF1 regulation networks (Figure 5B; Table S1). Fifty-four genes including the well-known −Cu_Up genes are induced by Cu deficiency in the wild type and *citf1-1*, but not in the *spl7-1*, suggesting that their upregulation by −Cu is under the control of SPL7, but not of the CITF1. Correspondingly, ninety-six genes are regulated negatively only by SPL7. Similarly, we identified 41 and 77 genes which are upregulated and downregulated respectively in a CITF1 dependent fashion. Meanwhile, we found that 81 genes are responsive to Cu deficiency under the control of both SPL7 and CITF1 (Table S1).

From the transcriptome data, we found that the expression of Cu-economy genes is controlled specifically by SPL7. It is well-known that Cu-miRNAs (miR397, miR398, miR408 and miR857) negatively regulate the expression of Cu-economy genes *CSDs*, *CCS*, *LACs,* and *ARPN* [12,15,16,17]. In support of this, our transcriptome data also show the opposite expression patterns between Cu-miRNAs and Cu-economy genes. Consistent with the previous reports [12,21], the expression of *FSD1* is activated significantly by Cu deficiency, further supporting that FSD1 compensates for the lack of CSDs when Cu availability is limiting in cells. SPL7 also controls the expression of many Cu translocation genes which are not affected by CITF1, such as *YSL2*, *YSL3*, *CCH* and *COPT1* (Table S2). Thus, SPL7 may control the Cu distribution between tissues or organs.

Cu is required for ethylene perception since Cu acts as a cofactor of ethylene receptors [22]. Among those genes regulated by CITF1, *EIL2* (*ETHYLENE-INSENSITIVE3-like 2*) and *ERF042* (*ETHYLENE-RESPONSIVE TRANSCRIPTION FACTOR 042*) encodes transcription factors involved in ethylene signaling transduction (Table S2). Therefore, EIL2 and ERF042 may function as the nodes in the crosstalk between Cu signaling and ethylene signaling. It was reported that CITF1 transcription is responsive to jasmonic acids (JA) treatment and involved in regulation of JA-synthesis genes in anthers [19,23]. The expression levels of JA-synthesis genes were retrieved (Table S3). We observed that the expression of *LOX3*, *LOX4*, *AOS*, *AOC1*, *AOC2,* and *AOC3* was suppressed by Cu deficiency, and their expression levels were lower in both *spl7-1* and *citf1-1* than in the wild type. Therefore, SPL7 and CITF1 mediate the interaction between Cu signaling and JA synthesis.

Recently, it was reported that SPL7 mediates the copper-deficiency response in the presence of high nitrogen in *Arabidopsis thaliana* [24]. Our data suggest that the nitrogen signaling is also affected by SPL7 under Cu-deficiency conditions. The transcript levels of *NRT2.7*, encoding a tonoplast nitrate transporter [25], were upregulated under Cu deficiency in roots, dependently on SPL7 (Table S2; Figure 5C). Similarly, *CLE2* was also induced by Cu deficiency in roots, which is involved in expansion of plant root systems in a nitrogen-dependent manner [26]. Therefore, it is likely that *NRT2.7* and *CLE2* are involved in the crosstalk between Cu signaling and nitrogen signaling. Interestingly, we found that SPL7 and CITF1 coregulate Cu-deficiency-responsive genes including *COPT2*, *FRO4*, *FRO5,* and *MT1C* (*Metallothionein 1C*) (Table S2; Figure 5C). Taken together, our data suggest that SPL7 and CITF1 not only coregulate Cu-deficiency signaling pathway, but also have functions independent of each other in the Cu-deficiency response.

### 2.5. CITF1 and SPL7 Directly Bind to the Promoters of Cu Uptake Genes

**Figure 6:**
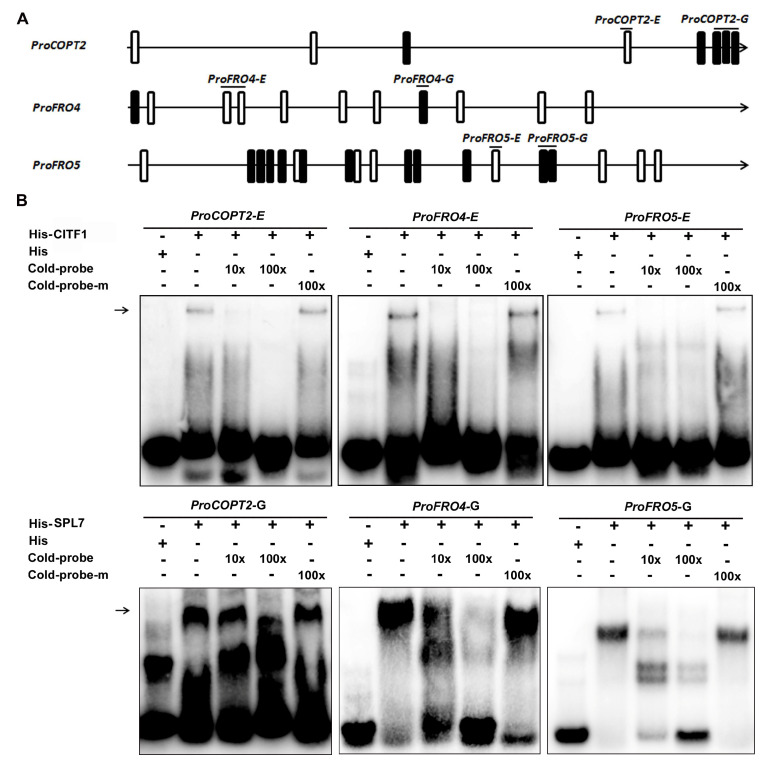
Both SPL7 and CITF1 bind to the promoters of *COPT2*, *FRO4,* and *FRO5*. (**A**) GTAC-boxes and E-boxes are indicated in the promoters. (**B**) EMSAs. Recombinant proteins indicated were incubated with the biotin-labeled probes. Biotin-probe, biotin-labeled probe; cold-probe, unlabeled probe; cold-probe-m, unlabeled mutated probe with mutated GTAC-boxes or E-boxes.

Given that the transcript abundance of *COPT2*, *FRO4,* and *FRO5* is coregulated by CITF1 and SPL7, we proposed that *COPT2*, *FRO4* and *FRO5* are the direct targets of SPL7 and CITF1. Generally, bHLH transcription factors recognize an E-box [27] and SPL7 recognizes a GTAC box. Sequence analysis indicated that there are several E-boxes and GTAC boxes in the promoters of *COPT2*, *FRO4,* and *FRO5* (Figure 6A), implying their interactions with CITF1 and SPL7. To investigate this hypothesis, we conducted EMSAs. The 6×His-tagged CITF1 was expressed in and purified from Escherichia coli. When His-CITF1 was incubated with the biotin-labeled *pCOPT2-E* probe with an E-box, His-CITF1 could bind to it, and this binding could be competed by the unlabeled *pCOPT2-E*, but not the mutation version *pCOPT2-mE*. In contrast, His-CITF1 could not bind to the promoter of *COPT1* (Figure S5). This suggests that CITF1 directly binds to the promoter of *COPT2* (Figure 6B). Similarly, we also confirmed that CITF1 binds to the promoters of *FRO4* and *FRO5* (Figure 6B). Meanwhile, we performed EMSAs with His-SPL7 and the probes of *COPT2*, *FRO4,* and *FRO5* with GTAC boxes, finding that SPL7 also directly binds to the promoters of *COPT2*, *FRO4,* and *FRO5*, but not the promoter of *COPT1* (Figure 6B and Figure S5).

**Figure 7:**
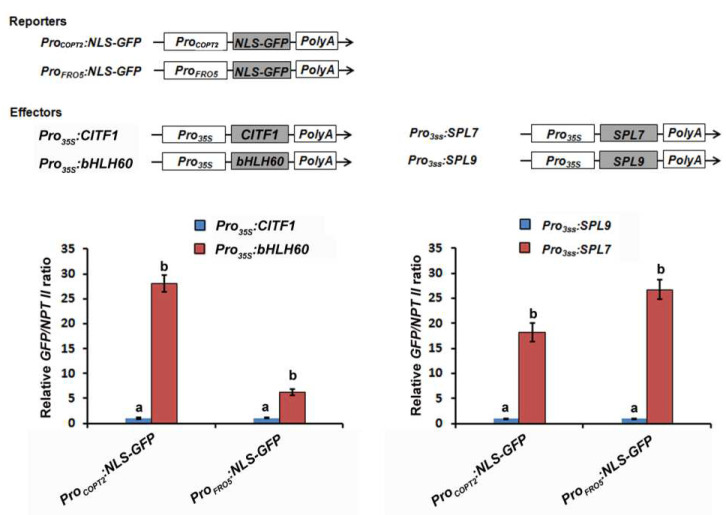
Activation of *COPT2* and *FRO5* promoters by SPL7 and CITF1. The *COPT2* and *FRO5* promoters were used to drive the NLS-GFP as the reporters. *bHLH60*, *CITF1*, *SPL7*, and *SPL9* respectively driven by the CaMV 35S promoter were used as the effectors. The bHLH60 and SPL9 were used as negative control effectors. The *GFP/NPT II* ratio represents the *GFP* transcript abundance relative to the internal control *NPT II* transcript. Data represent means ± SD (*n* = 3). Different letters indicate statistically significant differences of the mean values (*p* < 0.01).

To further investigate the regulation of *COPT2*, *FRO4,* and *FRO5* by SPL7 and CITF1, we used the reporter-effector transient expression system mentioned above. The promoters of *COPT2* and *FRO5* were respectively fused with the NLS-GFP reporter gene as the reporters (Figure 7). *Pro_35S_:bHLH60*, *Pro_35S_:SPL9*, *Pro_35S_:CITF1* and *Pro_35S_:SPL7* were used as the effectors. When *Pro_COPT2_:NLS-GFP* was used as the reporter, both *Pro_35S_:CITF1* and *Pro_35S_:SPL7* effectors considerably promoted the expression of GFP compared with the control effectors *Pro_35S_:bHLH60* and *Pro_35S_:SPL9*. Similar results were also obtained when *Pro_FRO5_:NLS-GFP* was used as the reporter. Taken together, our data suggest that both SPL7 and CITF1 directly bind to and activate the promoters of *COPT2*, *FRO4,* and *FRO5*.

## 3. Discussion

Plants have evolved sophisticated mechanisms to maintain Cu homeostasis. It has been documented that plants respond to Cu deficiency through upregulation of a number of genes involved in the acquisition of Cu from the extracellular environment, its transport into the cells, and its metabolism. It is well known that SPL7 is a master regulator of Cu-deficiency response in *Arabidopsis*. In this study, we report that SPL7 regulates the expression of *CITF1* by directly associating with its promoter, and both of them are required for the activation of the Cu uptake system under Cu deficiency conditions.

bHLH proteins are a superfamily of transcription factors and important regulatory components in transcriptional networks in plants. There are 162 bHLH members in *Arabidopsis* [28]. Many bHLH transcription factors have been characterized to control the expression of genes involved in metal homeostasis [29,30,31,32,33,34,35,36,37,38,39,40,41,42]. CITF1 was previously identified as a Cu-deficiency-responsive transcription factor regulating Cu homeostasis [19]. Here, we further established the functions of CITF1 in the Cu-homeostasis-signaling pathway. Cu deficiency leads to a significant increase in the *CITF1* transcript abundance [12,15,19]. When the function of CITF1 is lost, the expression of its target genes (*COPT2*, *FRO4,* and *FRO5*) is reduced (Figure S2) [19]. Lower expression levels of *COPT2*, *FRO4,* and *FRO5* can lead to decreased solubility and transport of Cu into root epidermal cells [12,43]. The overexpression of *CITF1* results in the increased expression of Cu uptake genes and elevated Cu concentration (Figure 1C,D). We also provide evidence that CITF1 directly regulates the expression of Cu uptake genes (*COPT2*, *FRO4,* and *FRO5*) in response to Cu deficiency (Figure 6 and Figure 7). Thus, the maintenance of Cu homeostasis requires the appropriate expression levels of *CITF1*.

We confirmed that there is a direct linkage between SPL7 and CITF1 because SPL7 directly binds to and activates the promoter of *CITF1* (Figure 2). However, it remains unknown how the transcription of *CITF1* is suppressed under Cu-sufficiency conditions. We assume that under Cu-sufficiency conditions, either a negative regulator which targets the *CITF1* promoter is activated, or its positive regulator SPL7 is inactivated, or both. From our transcriptome data, we identified many transcription factors which are downregulated under Cu-deficiency conditions, such as *MYB87*, *WRKY46*, *ZAT7,* and *ERF021* (Table S1). Further investigation is required to clarify whether these transcription factors negatively regulate the expression of *CITF1* under Cu-deficiency conditions. Given that *CITF1* is transcriptionally activated in response to Cu deficiency, it is expected that SPL7 is more active and (or) more stable under Cu-deficiency conditions than under Cu-sufficiency conditions. Therefore, further investigation on the SPL7 activity in response to Cu deficiency will help us understand the regulation mechanism of Cu homeostasis.

Plants can sense the internal Cu status and reprogram the transcription of Cu-homeostasis-associated genes which are responsible for Cu uptake and allocation. Although the *Arabidopsis* Cu sensor is not identified yet, SPL7 is a major regulator upstream of the Cu-deficiency-responsive signaling network. The interaction between the SPL7 protein and the promoter of *CITF1* also provides evidence that the Cu-homeostasis regulation mediated by SPL7 is partly dependent on CITF1. Our genetic evidence suggests that CITF1 acts downstream of SPL7 to take part in the regulation of Cu homeostasis (Figure 3). Although CITF1 is not induced by Cu deprivation in the *spl7-1*, the *citf1-1 spl7-1* double mutants displayed severer Cu-deficiency symptoms than the *spl7-1* (Figure S4D,E) [19]. It implies that the basal *CITF1* expression is required for the maintenance of Cu homeostasis. In support of this, we found that some genes are only impacted by CITF1, not by SPL7, under −Cu conditions (Table S2), which indicates the functions of CITF1 are independent of SPL7 in Cu homeostasis. The lower levels of Cu uptake genes in the *citf1-1 spl7-1* than in the *citf1-1* and *spl7-1* suggest that SPL7 and CITF1 have additive roles in regulating *COPT2*, *FRO4,* and *FRO5* (Figure 4). Our data also provide evidence that both SPL7 and CITF1 directly activate their expression (Figure 6 and Figure 7). Additionally, Yan et al., (2017) reported that the *citf1-1 spl7-1* plants are unable to grow in soil under Cu-sufficient conditions, but we did not observe this phenomenon. It is possible that the difference of soil components accounts for the different phenotypes in soil.

The regulatory network mediated by SPL7 was well established in *Arabidopsis* [12,15,44]. Our transcriptome data show that, among the genes responsive to Cu deficiency, 27% and 23% are affected by SPL7 and CITF1, respectively (Figure 5B). Bernal et al. [12] identified 206 SPL7-dependent Cu-deficiency-responsive genes and we identified 231. Most of them are shared in both studies. Therefore, we feel confident that this study captured a large number of transcriptional responses controlled by SPL7 and CITF1. By transcriptome analysis, we identified CITF1-dependent and -independent Cu-deficiency-responsive genes. The expression of several ethylene signaling genes is affected only by CITF1 (Table S2). Our transcriptome data show that *COPT2*, *FRO4* and *FRO5* are coregulated by CITF1 and SPL7, and we show evidence that CITF1 and SPL7 target different cis elements in the promoters of *COPT2*, *FRO4,* and *FRO5*. It was reported that CITF1 cooperates with SPL7 to regulate JA synthesis in flowers by affecting the expression of *LOX3*, *LOX4*, *AOS*, *AOC1*, *AOC2*, *AOC3,* and so on [19]. A recent study also uncovered that jasmonate induces alternative splicing of CITF1 [23]. In our root transcriptome, the expression of *LOX3*, *LOX4*, *AOS*, *AOC1*, *AOC2,* and *AOC3* in the *spl7-1* and *citf1-1* is lower than in the wild type under Cu sufficient conditions (Table S3), implying that SPL7 and CITF1 participate in the regulation of JA synthesis in roots. Further investigation is required to establish if there is a direct connection between CITF1 and the JA-synthesis-associated genes. Unexpectedly, we found that some Fe-deficiency-responsive genes are positively regulated by both SPL7 and CITF1 (Table S1), inconsistent with the previous reports [12,44]. In our experiments, EDTA was used to chelate iron in the 1/2 MS media for plant growth. It was reported that the medium containing EDTA becomes iron deficient because iron becomes unchelated and forms an unavailable precipitate as EDTA is oxidized by light [45]. It is likely that the Fe-deficient environment caused by EDTA oxidation resulted in the unexpected upregulation of several Fe-deficiency-responsive genes.

CITF1 and SPL7 are two key transcriptional regulators that control the expression of genes involved in a range of biological activities important for responding to Cu-deficient conditions. Our work sheds light on a new regulatory mechanism driving the root high affinity Cu uptake system in plants, and unravels the essential role of the CITF1 transcription factor directly stimulating Cu uptake gene expression during the Cu-deficiency responses. Further elucidating the interplay among genes in the CITF1-SPL7 network should provide much-needed insights into the mechanism by which plants adapt to Cu deficiency.

## 4. Materials and Methods

### 4.1. Plant Materials and Growth Conditions

*Arabidopsis thaliana* ecotype Col-0 was used as wild type. The *Arabidopsis thaliana* T-DNA insertion lines SALK_073160 (*citf1-1*) [19] and SALK_093849 (*spl7-1*) [12] were obtained from the *Arabidopsis* Biological Resource Center.

Seeds were surface-sterilized with 75% ethanol for 15 min and then washed three times with double-distilled water. After plated on 1/2 MS media, plates stayed for 2 d at 4 °C before shifting in greenhouse. Cu-sufficient (+Cu) medium is 1/2 MS medium with 1% sucrose, 0.7% agar and 1 μM CuSO_4_ at pH 5.8, and Cu-deficient (−Cu) medium is the same without CuSO_4_. In all experiments on medium, contaminant metals from agar were removed by washing 3 times with 10 mM EDTA for 12 h, then 6 times with ultrapure water. Plates were placed in a culture room at 22 °C under a 16 h light/8 h dark photoperiod. For phenotype analysis, three biological replicates with each containing fifteen plants were conducted.

### 4.2. Plasmid Construction and Plant Transformation

The seamless cloning kit (Beyotime, Shanghai, China) was used for plasmid construction. One microgram genomic DNA from *Arabidopsis* was used as the template for amplification of the upstream regulatory promoter sequences. For complementation of the citf1-1 mutant, a 1.4 k genomic fragment upstream of *CITF1* translation start site was PCR-amplified from Col-0 and used to drive the HA-tagged CITF1. For the generation of *CITF1-OE* or *spl7-1/CITF1-OE* plants, the CaMV 35S promoter was used to drive the HA-tagged CITF1 in the *pOCA30* binary vector. For the generation of *Pro_CITF1_:GUS* plants, a 1.4 k genomic fragment upstream of *CITF1* translation start site was fused with the *GUS* reporter in the *pOCA28* binary vector. For the construction of reporters, the corresponding promoters of *CITF1*, *COPT2,* and *FRO5* were respectively fused with the *NLS-GFP* sequence in the *pOCA28* vector [35]. For the construction of effectors, *CITF1*, *bHLH60*, *SPL7,* and *SPL9* were inserted respectively between the CaMV 35S promoter and poly(A) in the pOCA30 vector. Primers used for these constructs are listed in Table S4. All constructs were introduced into Agrobacterium tumefaciens strain EHA105.

*Arabidopsis* transformation was conducted by the floral dip method [46]. *Pro_35S_:HA-CITF1* was transformed into the wild type and *spl7-1* plants. *Pro_CITF1_:HA-CITF1* was transformed into the *citf1-1* plants. *Pro_CITF1_:GUS* was transformed into the wild type plants. Transgenic plants were selected with the use of 50 mg/L kanamycin.

### 4.3. Transient Expression Assays

The plasmids (*Pro_35S_:bHLH60*, *Pro_35S_:SPL9*, *Pro_35S_:CITF1*, *Pro_35S_:SPL7*, *Pro_CITF1_:NLS-GFP*, *Pro_COPT2_:NLS-GFP,* and *Pro_FRO5_:NLS-GFP*) were transformed into *Agrobacterium* tumefaciens strain EHA105. Agrobacterial cells were infiltrated into leaves of *Nicotiana benthamiana* by the infiltration buffer (0.2 mM acetosyringone, 10 mM MgCl_2_, and 10 mM MES, pH 5.6). In the transient expression assays, the final optical density at 600 nm value was 0.5 for reporters and effectors. After infiltration, plants were kept in dark for two days. The infiltrated leaves were harvested and used for RNA extraction. The transcript abundance of GFP was normalized to NPT II in the reporter vector.

### 4.4. EMSA

We used the Chemiluminescent EMSA Kit (Beyotime, Shanghai, China) following the manufacturer’s protocol to do EMSA Assays. The SBP domain of SPL7 (from amino acid position 122 to 228) and the full-length CITF1 were respectively fused in frame with the 6×His tag in the expression vector pET-28a. Primers used for these constructs are listed in Table S4. The resulting plasmids were introduced into Escherichia coli BL21 (DE3) for recombinant His-SPL7 and His-CITF1 protein expression. Cultures were grown overnight at 28 °C with 0.5 mM isopropyl β-D-1-thiogalactopyranoside. Proteins were extracted and purified by the His-tag Protein Purification Kit (Beyotime, Shanghai, China) following the manufacturer’s protocol. The DNA fragments used for probes were synthesized and biotin was labeled to the 5′ terminal of DNA. Biotin-unlabeled fragments of the same sequences or mutated sequences were used as competitors, and the negative control was His protein alone.

### 4.5. Cu Measurement

Plants were grown in soil for 4 weeks and leaves were harvested for the measurement of Cu concentration. Leaves were rinsed with deionized water and dried at 65 °C for 3 d. About 100 mg dry weight was wet-ashed with 5 mL of 11 M HNO_3_ and 1 mL of 12 M HClO_4_ for 20 min at 220 °C. Each sample was diluted to 16 mL with 18 MΩ water and Cu concentration was analyzed on a Thermo SCIENTIFIC ICP-MS (iCAP6300), Waltham, MA, USA. Reference standards were used for quantification. Each biological sample was measured three times and a mean value was calculated.

### 4.6. Gene Expression Analysis

Roots were collected from plants grown as described above and were frozen in liquid nitrogen. Roots were ground in liquid nitrogen to a fine powder. Total RNA was extracted by the Trizol reagent (Invitrogen, Carlsbad, CA, USA). One microgram of total RNA was used for oligo (dT)18 primed cDNA synthesis by the use of HiScript II Q RT. SuperMix for qPCR (+gDNA wiper) (Vazyme, Nanjing, China) according to the manufacturer’s protocol in a 20 μL volume. Prior to RT-qPCR analysis, primer and cDNA concentrations were optimized to reach the gene amplification efficiency of 100% ± 10%. Two microliters of 15-fold diluted cDNA were used in a 20 μL qPCR reaction on a Light-Cycler 408 real-time PCR machine (Roche, Basel, Switzerland). Ten microliters of AceQ Universal SYBR qPCR Master Mix (Vazyme, Nanjing, China) was used for a 20 μL qPCR reaction. The PCR program consisted of one cycle (95 °C, 4 min), 40 cycles (95 °C, 10 s; 60 °C, 20 s; 72 °C, 15 s), and one cycle (72 °C, 5 min), followed by a melting curve program (55 to 90 °C in increasing steps of 3 °C). Real-time PCR experiments were conducted using three independent experiments with each containing three technical replicates. The relative expression of genes was normalized to *ACT2* and *TUB2*. All primers used for qRT-PCR are listed in Table S4.

### 4.7. High-Throughput Sequencing of mRNA, and Differential Gene Expression Analysis

For transcriptome analysis, roots of wild-type, *citf1-1,* and *spl7-1* plants grown on +Cu or −Cu 1/2 MS medium for 7 days were used for RNA extraction. Three replicates of the whole roots analysis were performed. Total RNA of each sample was extracted according to the instruction manual of the TRlzol Reagent (Invitrogen, Carlsbad, CA, USA). RNA integrity and concentration were checked using an Agilent 2100 Bioanalyzer (Agilent Technologies, Inc., Santa Clara, CA, USA). The mRNA was isolated by NEBNext Poly (A) mRNA Magnetic Isolation Module (NEB, E7490). The cDNA library was constructed following the manufacturer’s instructions of NEBNext Ultra RNA Library Prep Kit for Illumina (NEB, E7530) and NEBNext Multiplex Oligos for Illumina (NEB, E7500). In briefly, the enriched mRNA was fragmented into approximately 200nt RNA inserts, which were used to synthesize the first-strand cDNA and the second cDNA. The double-stranded cDNA was performed end-repair/dA-tail and adaptor ligation. The suitable fragments were isolated by Agencourt AMPure XP beads (Beckman Coulter, Brea, CA, USA), and enriched by PCR amplification. Finally, the constructed cDNA libraries were sequenced on an Illumina sequencing platform.

Transcript abundance was concluded to increase/decrease under Cu deficiency for a gene when arithmetic means of transcript abundance differed by a factor of at least 2. Changes in transcript levels were concluded to be dependent on SPL7 if log_2_ FC (wild type −Cu versus *spl7-1* −Cu) > 1 for log_2_ FC (wild type −Cu versus wild type +Cu) > 1, and log_2_ FC (wild type −Cu versus *spl7-1* −Cu) < 1 for log_2_ FC (wild type −Cu versus wild type +Cu) < −1. The CITF1 dependent transcripts were obtained by a similar filtration.

## Data Availability

The raw sequencing data are stored in NCBI under the accession number of PRJNA665579 (https://www.ncbi.nlm.nih.gov/sra/PRJNA665579) accessed on 10 October 2020.

## Supplementary Materials

The following supporting information can be downloaded at: https://www.mdpi.com/article/10.3390/ijms23137239/s1.
