# Supplementary material for: CITF1 Functions Downstream of SPL7 to Specifically Regulate Cu Uptake in Arabidopsis
Source: Int J Mol Sci. 2022 Jun 29;23(13):7239. doi: 10.3390/ijms23137239 (PMC9266912; doi:10.3390/ijms23137239)
Supplement: Supplementary file 1 [file ijms-23-07239-s001.zip › ijms-1777439-supplementary/Supplementary Figures.pdf]

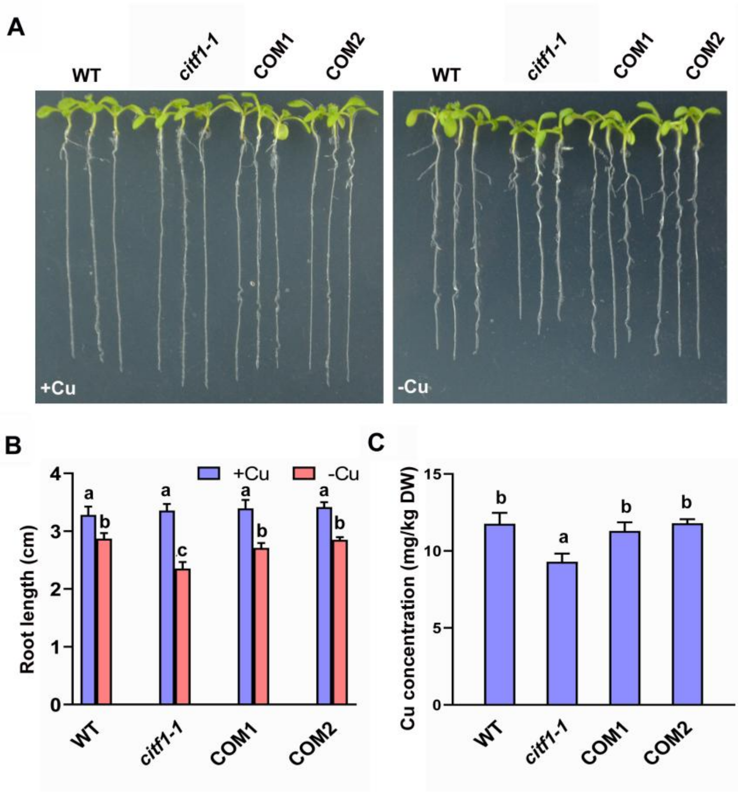

Figure S1. Loss-of-function of *CITF1* impairs Cu homeostasis .

(A) Phenotypes of seedlings. Seedlings grown on +Cu or –Cu medium for 7 days are shown.

(B) Root length. Seven-day-old seedlings were analyzed. Three biological replicates were conducted. Each biological replicate contained at least 15 plants. Data represent arithmetic means ± SD (n = 3 biological replicates, each of which contained at least 15 plants). Different letters above each bar indicate statistically significant differences (ANOVA, $P < 0.01$).

(C) Cu concentration. Leaves from plants grown in soil for 3 weeks were used for Cu measurement. Data represent means ± SD (n = 3 biological replicates, each of which contained 100 mg pooled dry leaves). Different letters above each bar indicate statistically significant differences (ANOVA, $P <$ 0.01).

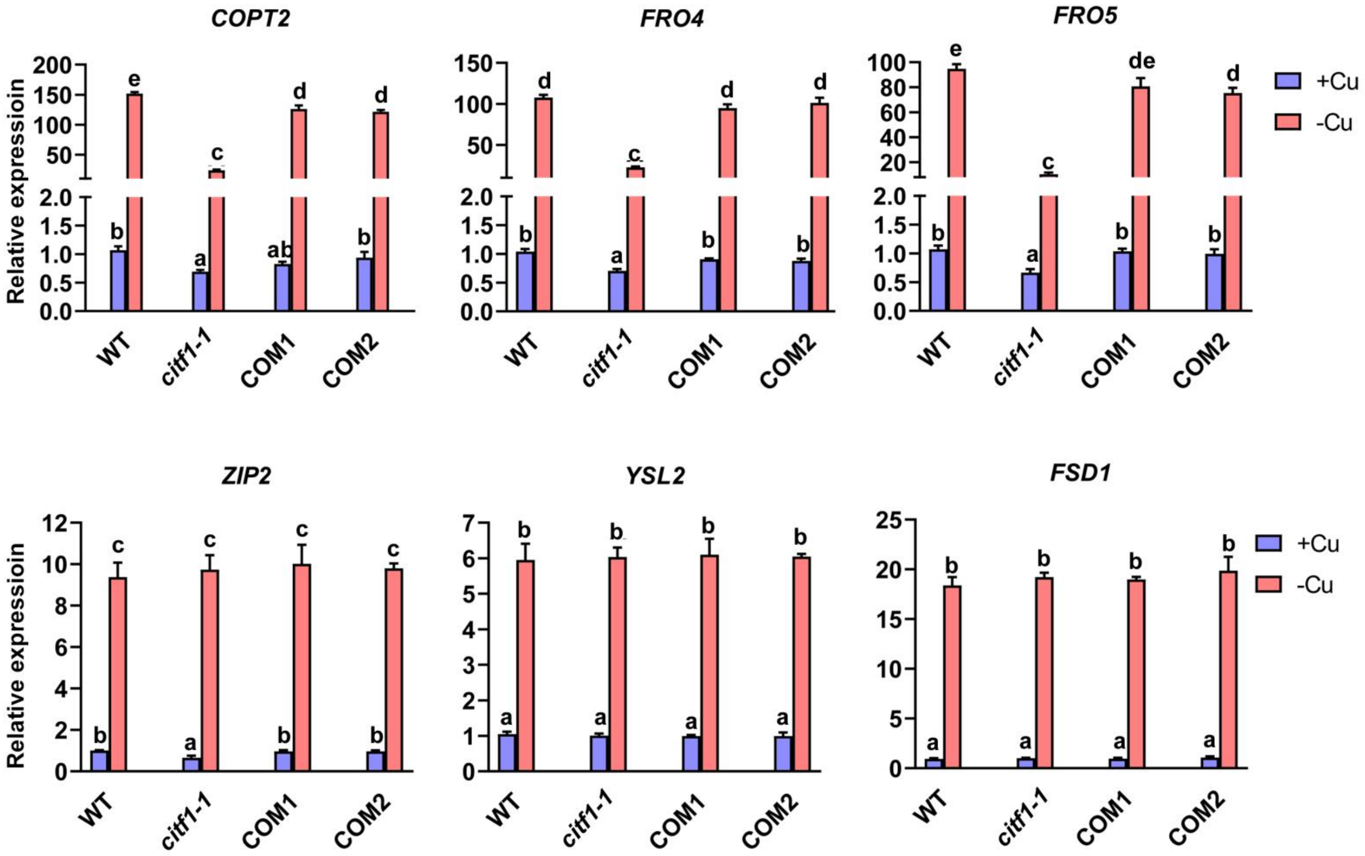

Figure S2. Expression of Cu-deficiency responsive genes in *citf1-1*.

Expression of Cu-deficiency responsive genes. Seedlings were grown on +Cu or –Cu medium for 7 days. Roots were used for RNA extraction. Data represent means ± SD (*n* = 3 technical repeats from one representative experiment; three independent experiments were performed). Different letters indicate statistically significant differences of the mean values (P < 0.01)

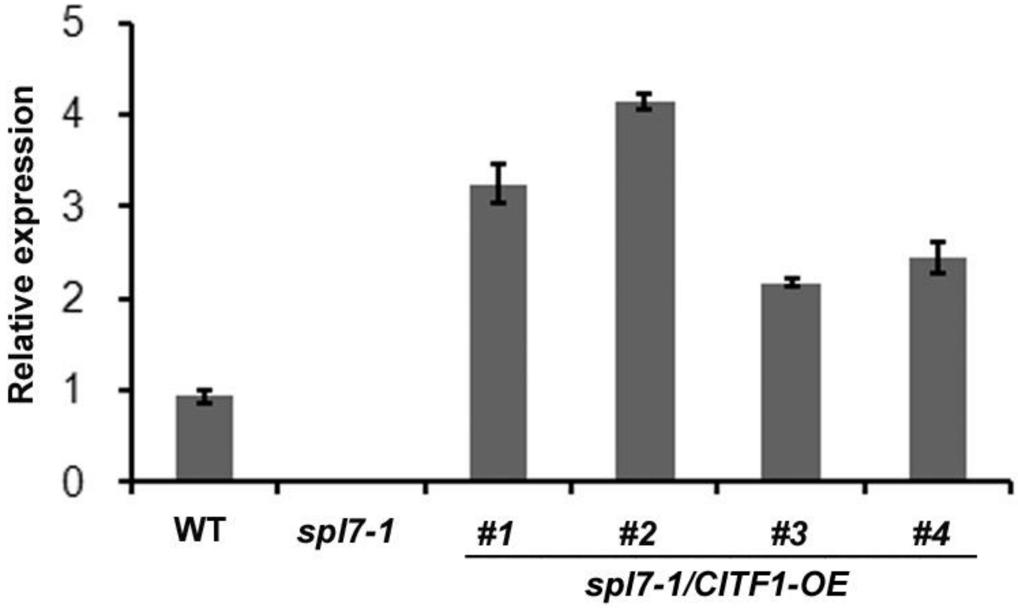

Figure S3. Identification of *spl7-1/CITF1-OE* lines.

Seven-day-old roots grown on –Cu medium were harvested and used for protein extraction. Roots were used for RNA extraction. Data represent means ± SD (*n* = 3 technical repeats from one representative experiment; two independent experiments were performed).

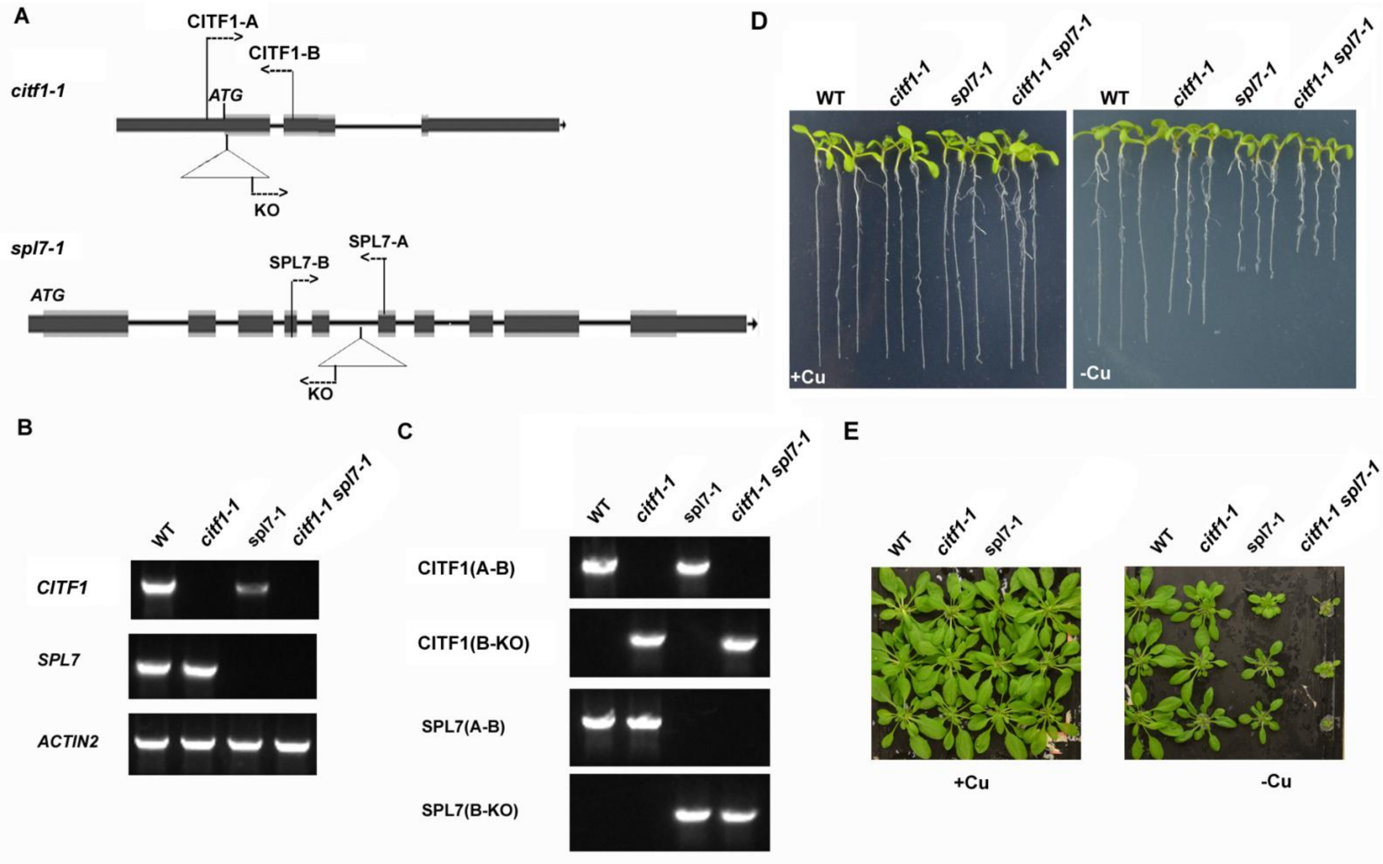

Figure S4. Identification of *citf1-1 spl7-1* mutants.

(A) Exon-intron structure and T-DNA insertion sites in the *citf1-1* and *spl7-1* mutants. A triangle indicates the T-DNA insertion position. The arrows indicate the primers used in (C).

(B) Transcripts of *CITF1* and *SPL7*. Roots were used for RNA extraction and reverse transcription reaction.

(C) Identification of *citf1-1 spl7-1* mutant. Genomic DNA was used for PCR.

(D) One-week-old seedlings. Plants were grown on +Cu or –Cu solid medium.

(E) Five-week-old seedlings. Two-week-old seedlings grown on +Cu or –Cu solid medium were shifted to liquid solution for three weeks.

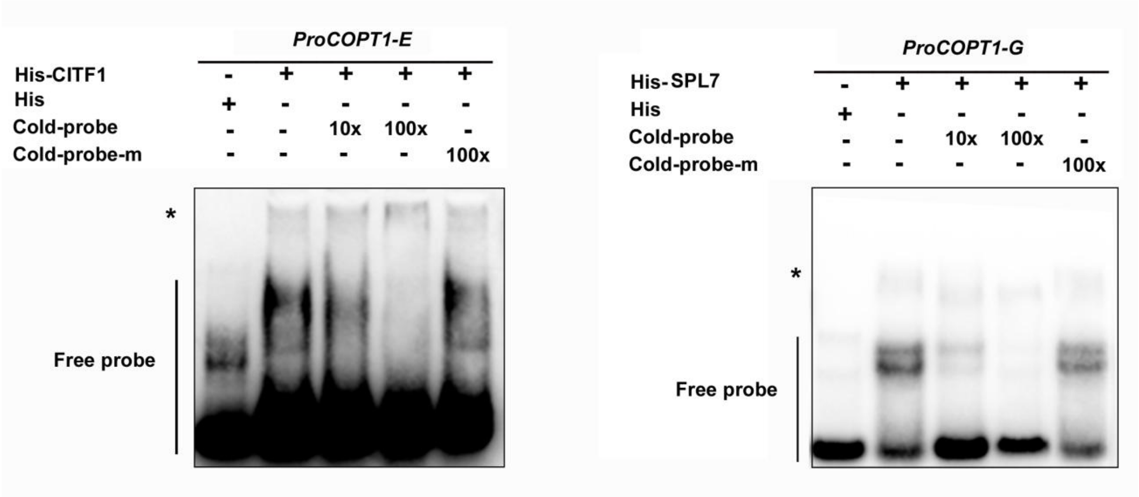

Figure S5. SPL7 and CITF1 do not bind to the promoter of *COPT1*.

EMSA showing that SPL7 and CITF1 could not bind to the promoter of *COPT1*. Either His-SPL7 or 6xHis was incubated with the biotin-labeled probes. Biotin-probe, biotin-labeled probe; cold-probe, unlabeled probe; cold-probe-m, unlabeled mutated probe with a mutated GTAC-box or E-box. * indicates non-specific bindings.
